# A Novel Approach for Mining Polymorphic Microsatellite Markers *In Silico*

**DOI:** 10.1371/journal.pone.0023283

**Published:** 2011-08-10

**Authors:** Joseph I. Hoffman, Hazel J. Nichols

**Affiliations:** 1 Department of Animal Behaviour, University of Bielefeld, Bielefeld, North Rhine-Westphalia, Germany; 2 Department of Zoology, University of Cambridge, Cambridge, Cambridgeshire, United Kingdom; Lund University, Sweden

## Abstract

An important emerging application of high-throughput 454 sequencing is the isolation of molecular markers such as microsatellites from genomic DNA. However, few studies have developed microsatellites from cDNA despite the added potential for targeting candidate genes. Moreover, to develop microsatellites usually requires the evaluation of numerous primer pairs for polymorphism in the focal species. This can be time-consuming and wasteful, particularly for taxa with low genetic diversity where the majority of primers often yield monomorphic polymerase chain reaction (PCR) products. Transcriptome assemblies provide a convenient solution, functional annotation of transcripts allowing markers to be targeted towards candidate genes, while high sequence coverage in principle permits the assessment of variability *in silico*. Consequently, we evaluated fifty primer pairs designed to amplify microsatellites, primarily residing within transcripts related to immunity and growth, identified from an Antarctic fur seal (*Arctocephalus gazella*) transcriptome assembly. *In silico* visualization was used to classify each microsatellite as being either polymorphic or monomorphic and to quantify the number of distinct length variants, each taken to represent a different allele. The majority of loci (*n* = 36, 76.0%) yielded interpretable PCR products, 23 of which were polymorphic in a sample of 24 fur seal individuals. Loci that appeared variable *in silico* were significantly more likely to yield polymorphic PCR products, even after controlling for microsatellite length measured *in silico*. We also found a significant positive relationship between inferred and observed allele number. This study not only demonstrates the feasibility of generating modest panels of microsatellites targeted towards specific classes of gene, but also suggests that *in silico* microsatellite variability may provide a useful proxy for PCR product polymorphism.

## Introduction

Microsatellites, also known as Short Tandem Repeats (STRs), Simple Sequence Repeats (SSRs) or Variable Number Tandem Repeats (VNTRs) are DNA segments comprising tandemly repeated motifs of 1–6 nucleotides. They are ubiquitous in eukaryotic and prokaryotic genomes, present in both coding and non-coding regions, and also have a high enough mutation rate (between 10^−3^ and 10^−4^ mutations per gamete per generation) to generate and maintain extensive length polymorphism [1], [2]. This makes them powerful genetic markers for a variety of applications ranging from the determination of parentage and other genetic relationships to genetic mapping [3], [4]. However, a major drawback of microsatellites is that for most species they need to be developed *de novo*, a process that is often costly and protracted [5].

The most commonly used approach for developing microsatellites requires the construction of a partial genomic library enriched for repetitive motifs, cloning, hybridization to detect positive clones, plasmid isolation and Sanger sequencing followed by primer design and evaluation [5]. Although most of these steps involve relatively straightforward protocols, they can be time-consuming due to the frequent need for troubleshooting. Moreover, established protocols for enrichment and cloning can be highly inefficient. For example, the yield of positive clones typically averages only around 2–3% but can fall to as little as 0.03% [5]. This can be particularly problematic when attempting to isolate markers from species with genomes that are relatively depauperate in microsatellites such as many birds [6] and fungi [7].

Fortunately, recently developed next-generation sequencing platforms such as Roche's GS-FLX (454 Life Sciences, Branford, CT, USA) provide novel avenues for isolating microsatellites. A single 454 run is capable of generating around 400 Mb of sequence data, with individual reads long enough (up to 500 bp) to capture individual microsatellites along with enough flanking sequence to design PCR primers. Sequence generation on such a scale bypasses the need for enrichment because even a fraction of a 454 run can yield a sufficiently large number of random sequence reads to contain many thousands of microsatellites by chance [8]–[12]. Moreover, by directing 454 sequencing towards expressed genes (i.e. transcriptome sequencing), the possibility now exists to develop panels of microsatellites that are ideal markers for genes underlying phenotypic variation [13].

Given the clear advantages afforded by 454 sequencing, approaches based on this or other emerging hi-throughput technologies will inevitably supercede conventional microsatellite isolation protocols. However, it will continue to be necessary to design primers, optimize each primer pair for PCR and then test these for polymorphism in a sample of individuals of the study species. This is because not all primer pairs generate interpretable PCR products and a proportion of loci that amplify successfully are invariably monomorphic. The latter represent wasted effort, an issue that can be particularly acute in species with low genetic diversity where large numbers of loci often need to be evaluated in order to obtain just a handful of informative markers. An example comes from the endangered Hawaiian monk seal, in which Shultz et al. [14] developed 163 microsatellite loci in order to obtain just 17 that were polymorphic. For systems such as this, but also to bring about more general improvements in efficiency, it would therefore be desirable to develop and evaluate potential approaches for pre-screening microsatellites for polymorphism *in silico*.

Just such an opportunity is provided by a large body of 454 sequence data recently generated for the Antarctic fur seal, *Arctocephalus gazella* [15]. A long-term genetic study of this colonially breeding polygynous pinniped species at Bird Island, South Georgia [16] has identified remarkably consistent relationships between heterozygosity measured at 9–76 microsatellite loci and a variety of important fitness traits, from male reproductive success through body size to attractiveness [17]–[20]. However, despite several candidate genomic regions being identified through the mapping of individual microsatellites to the dog (*Canis lupis familiaris*) genome (seals and dogs diverged approximately 44 million years ago [21] and the canine genome is relatively well annotated), the underlying genes remain elusive [19]. Plausibly, most if not all of these could be immune-related, with reduced parasite loads and disease leading to greater longevity, enhanced growth, behavioural dominance and attractiveness. However, an alternative possibility is that heterozygosity might directly impact an individual's metabolism [22], allowing it to grow more quickly and to attain greater body size. Consequently, we 454 sequenced the skin transcriptome of this species [15], both to generate a preliminary genomic resource for pinnipeds in general and with a view towards identifying markers within suitable candidate genes.

Our experimental approach comprised two main stages (see Materials and Methods for further details). First, 454 sequencing was conducted on pooled cDNA from twelve Antarctic fur seal individuals (six adult males, two adult females and four pups) selected to capture much of the allelic diversity present within the population [15]. The resulting reads were then assembled *de novo* into isotigs, each representing a collection of transcripts of a given gene, which were in turn functionally annotated by reference to the dog genome using Gene Ontology (GO) codes. We then used freeware to interrogate all of the isotig sequences for microsatellite repeat motifs. Finally, candidate markers were selected by filtering for a subset of isotigs containing microsatellites and with GO annotation terms related to immunity and growth.

In the second stage, we attempted to quantify the variability of each locus *in silico* and relate this to observed polymorphism when the locus was PCR amplified in a panel of 24 unrelated fur seals (note that these individuals were different from those used to generate the initial transcriptome assembly). To estimate levels of *in silico* variability, microsatellite-containing isotigs were visualized within the program Tablet [23]. This provides a graphical representation of each isotig in which the individual reads comprising it are shown aligned against the consensus sequence. Upon visual inspection, certain microsatellites appeared identical across multiple reads whereas others showed evidence of variation in the numbers of repeat units. We therefore quantified for a subset of loci the number of repeat units within each of the reads, allowing derivation of estimates of variability including the number of distinct motif length variants (each of which was taken to represent a different allele) and the number of reads differing from the consensus genotype.

In this manuscript, we evaluated a total of fifty primer pairs designed to PCR amplify putative microsatellites identified from the Antarctic fur seal transcriptome assembly. Our aims were twofold: (i) to generate a panel of microsatellites functionally linked to either immunity or growth; and (ii) to explore the relationship between *in silico* variability and PCR product polymorphism.

## Materials and Methods

### Sequence data and bioinformatic analysis

Library construction, 454 sequencing, assembly, annotation and mapping to the dog genome are described in detail by Hoffman [15]. Briefly, a normalized cDNA library derived from skin samples of twelve Antarctic fur seal individuals was sequenced on a Roche GS-FLX DNA sequencer (Roche Diagnostic), generating 1,443,397 reads of mean length of 286 bp. These were then assembled *de novo* using Roche Newbler assembler version 2.3 into 23,025 isotigs, which in turn clustered into 18,576 isogroups (different isotigs from a given isogroup can be inferred as alternative splice-variants). Mean isotig length was 854 bp and the average depth of coverage was 19.4×. Basic Local Alignment Search Tool (BLAST) similarity searches to the non-redundant database with an e-value threshold of 1e^−4^ produced matches for 10,825 isotig sequences (47.0%), with 76.9% of the top matches being to mammals and these most frequently comprising the dog. Restricting the BLAST search set to canine sequences, the majority of isotigs (*n* = 22,541, 97.9%) were also mapped to unique locations within the dog genome (NCBI Build 2, “Dog2.0”, dated 10 May 2005). A final set of BLAST searches against a subset of sequences with known Gene Ontology (GO) annotations recovered a total of 111,446 annotation terms.

### Microsatellite identification and selection

The program SSRIT [24] was used to identify isotigs containing perfect di-, tri- and tetranucleotide repeats with a minimum length of five repeat units. A total of 2271 loci were identified, 1871 (82.4%) of which comprised dinucleotides, 301 (13.3%) trinucleotides and 99 (4.4%) tetranucleotides (available via Dryad, doi: 10.5061/dryad.8268). These were located within 1939 different isotigs, of which 864 (44.6%) were functionally annotated and 1834 (94.6%) mapped to known regions in the dog genome. To target microsatellites residing within candidate immune or growth-related genes, we used a relational database to filter all of the isotigs for a subset with GO annotation terms containing the strings ‘immun’ or ‘growth’, recovering a total of 316 and 1132 isotigs (1.37% and 4.92%) respectively. Of these, 26 (8.23%) and 106 (9.36%) contained repetitive motifs respectively. Oligonucleotide primers were designed to amplify PCR products for a further subset that (i) contained sufficient flanking sequence on both sides of the repetitive motif to allow the design of both forward and reverse primers; (ii) had a minimum of 2× coverage of both the microsatellite and adjacent flanking regions; (iii) were BLAST annotated with respect to the nr database, and (iv) mapped to known regions within the dog genome. To avoid redundancy, we also avoided designing primers for more than one representative of any given isogroup. Primers were designed using the program Primer 3 [25] to amplify 100–250 bp products, to have a melting temperature (T_m_) as close as possible to 60°C and a maximum difference in T_m_ between the two primers of 3°C. A total of 50 primer pairs were designed. These comprised 13 and 27 pairs to amplify microsatellites residing within isotigs with immune and growth-related GO codes respectively (Agt1 to Agt13 and Agt14 to Agt40) plus a further 10 pairs developed to amplify loci selected on the basis of appearing highly variable *in silico* (Agt41 to Agt50, see Tables S1 and S2 for details).

### *In silico* quantification of microsatellite variability

**Figure 1:**
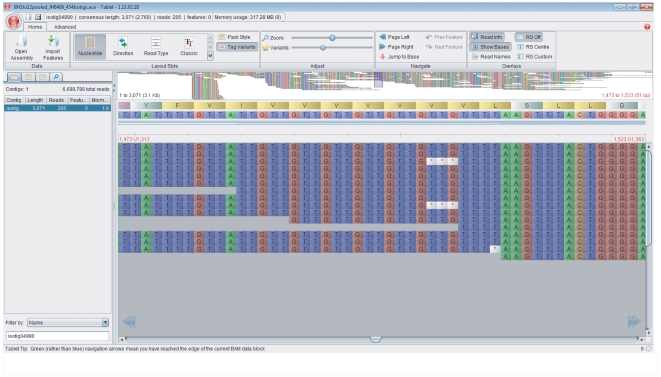
Screenshot of a polymorphic trinucleotide-repeat (TTG) microsatellite locus (Agt25) visualised *in silico* using the program Tablet [23]. The upper ‘overview window’ shows a scaled-to-fit summary of all of the reads comprising the isotig, while the main ‘display window’ shows the microsatellite and its immediate flanking regions visualised under a higher zoom. Within this window, 454 reads are shown aligned against the consensus sequence, with each read occupying a separate row. Individual bases are coloured according to nucleotide type and pad characters, introduced by Newbler to fill any gaps in the assembly, are represented by red star symbols against a light grey background. Two distinct motif length variants can be seen, comprising six and seven repeat units respectively. The same number of alleles was detected when the locus was PCR-amplified in 24 unrelated *A. gazella* individuals.

To derive *in silico* estimates of microsatellite variability, we visualized the sequence assembly within Tablet 1.11.02.18 [23]. This program can handle multiple input file formats and has several features that make it well-suited to exploring transcriptome assemblies (for an example screenshot showing a polymorphic microsatellite locus, see Figure 1). The main display window allows a single isotig to be viewed at a time and is navigable by means of scrolling and zooming functions. Within this window, each of the reads is shown aligned against the consensus sequence, with individual bases coloured according to nucleotide type and each read occupying a separate row under the ‘stacked format’ option. Pad characters, introduced by Newbler to fill any gaps in the assembly that arise for example where indels or length polymorphisms are present among the reads, are represented by star symbols. This format is amenable to quantifying the number of repeat motifs present within each of multiple reads at a given locus. This was done manually to generate *in silico* measures of variability for each of the microsatellite loci to be tested for PCR amplification, including the number of unique motif length variants (inferred to be different alleles) and the number of reads differing from the most common, consensus genotype.

### Tissue sampling

Skin biopsy samples were collected from 24 unrelated adult male Antarctic fur seal individuals during the austral summer of 2008/2009 at Bird Island, South Georgia (54°00′S, 38°02′W). These were taken using a remote biopsy dart system [26] and stored individually in 96% ethanol at −20°C. Samples were collected and retained under permits issued by the Department for Environment, Food and Rural Affairs (License number AHZ/2024A/2005/1) and in accordance with the Convention on International Trade in Endangered Species of Wild Fauna and Flora. All field procedures were approved by the British Antarctic Survey (reference number PEA 6).

### DNA extraction and genotyping

Total genomic DNA was extracted using an adapted Chelex 100 protocol [27] followed by phenol-chloroform purification [28]. All 50 selected primer pairs were tested for PCR amplification as described in detail by Hoffman and Amos [29]. The following PCR profile was used: one cycle of 120 s at 94°C, 45 s at 46°C, 50 s at 72°C; ten cycles of 30 s at 94°C, 45 s at 46°C, 50 s at 72°C; 25 cycles of 30 s at 89°C, 45 s at 48°C, 50 s at 72°C; and one final cycle of five minutes at 72°C. Primer pairs that failed to give a clear product under these conditions were also tested at a range of different annealing temperatures. PCR products were resolved by electrophoresis on standard 6% polyacrylamide sequencing gels and detected by autoradiography. Allele sizes were scored manually.

### Data analyses

Genepop [30] was used to calculate observed and expected heterozygosities and to test for deviations from Hardy-Weinberg equilibrium and for linkage disequilibrium. Null allele frequencies were calculated following Chakraborty [31] using the program Micro-checker [32]. To evaluate factors potentially influencing whether or not the observed PCR products were polymorphic, we constructed Generalized Linear Models (GLMs) within R [33]. Polymorphism was initially modeled as a binary response variable (1 = polymorphic, 0 = monomorphic) using a Binomial error structure. Restricting the dataset to loci that generated clearly interpretable polymorphic PCR products, we then constructed a second GLM in which polymorphism was expressed as the number of observed alleles and modeled using a Poisson error structure. The following predictor variables were fitted in both models: microsatellite length (measured as the number of repeat units comprising the shortest allele observed *in silico*), the number of reads differing from the consensus sequence and the total number of alleles observed *in silico* (all of which were fitted as continuous variables), the basis on which the marker was selected (as a factor with three levels: immune-related, growth-related or appearing highly variable) and motif (also as a three level factor: dinucleotide, trinucleotide or tetranucleotide). We additionally fitted *in silico* variability as a binary factor (0 = not variable, 1 = variable) in the first GLM. Using standard deletion-testing procedures [34], each term was progressively dropped from models unless doing so significantly reduced the amount of deviance explained (deviance is analogous to sums of squares in standard regression analysis). The change in deviance between full and reduced models was distributed as χ^2^ with degrees of freedom equal to the difference in degrees of freedom between the models with and without the term in question. For all models, distributions of standardized residuals about regressions were inspected to verify that they were approximately normally distributed.

## Results

Fifty primer pairs were designed to amplify microsatellites residing within transcripts selected either on the basis of GO codes related to immunity or growth (*n* = 13 and 27 respectively) or for appearing highly variable when visualized *in silico* (*n* = 10, see Tables S1 and S2 for details). The majority of primer pairs (*n* = 38, 76.0%) yielded PCR products that could be discriminated as either polymorphic or monomorphic in a sample of 24 unrelated Antarctic fur seal individuals (Table S2). Of these, 23 loci (60.5%) were polymorphic, although two could not be reliably scored due to the co-amplification of a second microsatellite. The remaining 21 loci possessed between 2 and 9 alleles each, with observed heterozygosity ranging from 0.042 to 0.917 (Table 1). Two of these loci (Agt5 and Agt49) deviated significantly from Hardy-Weinberg equilibrium (Table 1), although not significantly following Bonferroni correction for multiple tests [35]. Tests for linkage disequilibrium yielded 6 weakly significant *P* values (*P*<0.05) out of 210 pairwise comparisons, none of which remained significant following Bonferroni correction, indicating that the loci are unlikely to be physically linked. This is consistent with these transcript sequences mapping to 17 different chromosomes in the dog, with no more than 3 isotigs locating to any single chromosome (Table S1).

**Table 1:** Polymorphism characteristics of 21 microsatellite loci that amplified polymorphic and interpretable PCR products in 24 unrelated *Arctocephalus gazella* individuals.

Locus	Genbank accession number	Number of alleles	H_O_ a	H_E_ b	Null allele frequency^c^	HWE *P*-value^d^
Agt5	JF746971	2	0.053	0.235	0.626	0.012
Agt9	JF746972	2	0.125	0.120	−0.032	1.000
Agt10	JF746973	3	0.417	0.377	−0.061	0.483
Agt13	JF746974	3	0.125	0.121	−0.025	1.000
Agt16	JF746975	2	0.167	0.156	−0.044	1.000
Agt20	JF746976	2	0.250	0.223	−0.067	1.000
Agt21	JF746977	6	0.625	0.785	0.103	0.242
Agt23	JF746978	2	0.042	0.042	−0.011	NA
Agt24	JF746979	7	0.727	0.778	0.022	0.513
Agt25	JF746980	2	0.042	0.042	−0.011	NA
Agt32	JF746981	4	0.875	0.668	−0.145	0.182
Agt38	JF746982	2	0.087	0.085	−0.022	1.000
Agt39	JF746983	5	0.739	0.728	−0.019	0.791
Agt41	JF746984	9	0.917	0.839	−0.055	0.595
Agt42	JF746985	5	0.333	0.420	0.105	0.181
Agt44	JF746986	2	0.208	0.191	−0.055	1.000
Agt45	JF746987	3	0.522	0.581	0.043	0.394
Agt47	JF746988	3	0.391	0.476	0.087	0.243
Agt48	JF746989	6	0.875	0.757	−0.083	0.786
Agt49	JF746990	5	0.417	0.621	0.186	0.012
Agt50	JF746991	9	0.833	0.715	−0.087	0.364

a Observed heterozygosity.

b Expected heterozygosity.

c Negative null allele frequency values are normal using Chakarborty's estimator [31] when the null allele frequency is close to zero and sample sizes are small [64].

d Hardy-Weinberg equilibrium *P*-values could not be calculated for loci indicated by ‘na’ due to only one individual carrying the second allele.

### Null alleles

A common problem with microsatellites is the presence of non-amplifying alleles, which usually result from a mutation (base substitution, insertion or deletion) in one or both of the primer binding sites [36]–[38]. Both of the loci that deviated significantly from Hardy-Weinberg equilibrium carried null alleles at high to moderate frequencies (0.626 and 0.186 for Agt5 and Agt49 respectively, Table 1). Consequently, we inspected the primer binding sites of these two microsatellites within the program Tablet [23] for nucleotide sequence variation. A Single Nucleotide Polymorphism (SNP) was detected in the binding site of the Agt5 reverse primer (T/C, minor allele frequency = 0.143, depth of coverage = 21 reads). SNPs were not found in either of the primer binding sites of locus Agt49, but the minimum depth of sequence coverage was lower for these regions (9× and 4× for the forward and reverse primer sites respectively).

### PCR conversion rates and allelic richness

The proportion of primer pairs yielding clearly interpretable and polymorphic PCR products was similar for microsatellites residing within immune and growth-related transcripts (30.8%, *n* = 4 and 33.3%, *n* = 9 respectively) but substantially higher for loci selected on the basis of high *in silico* variability (80.0%, *n* = 8). The number of observed alleles was lowest for immune-related transcripts (mean = 2.50±0.29 SE), intermediate for growth-related transcripts (mean = 3.56±0.67 SE) and highest for microsatellites that appeared highly variable *in silico* (mean = 5.25±0.94 SE). However, variation in allele number was not statistically significant overall (one way ANOVA, F_2,20_ = 2.53, *P* = 0.11).

### Predictors of PCR product polymorphism

In over three quarters of cases (29/38), microsatellite loci appearing variable *in silico* generated polymorphic PCR products and vice-versa (Fisher's exact test, *P* = 0.0032, see Table 2 for a breakdown). The exceptions were a single locus that was inferred to be monomorphic from the 454 data but which yielded polymorphic PCR products, and eight loci that appeared variable *in silico* but which generated monomorphic products. However, all but two of the latter had only 1–3 reads differing from the consensus sequence, raising the possibility that these could have been either sequencing errors or genuine but low-frequency alleles.

**Table 2:** Table summarizing consistency between *in silico* and PCR product polymorphism across 38 microsatellite loci.

		PCR products		
		Polymorphic	Monomorphic	Total
*In silico*	Polymorphic	22	8	30
	Monomorphic	1	7	8
	Total	23	15	38

**Figure 2:**
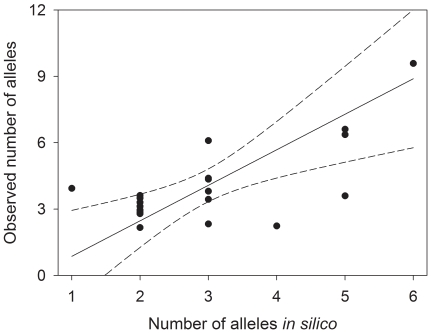
Relationship between the inferred number of alleles *in silico* and observed allele number for 21 polymorphic microsatellite loci. Shown are fitted values from a GLM controlling for the number of repeats and the number of reads differing from the consensus sequence. The solid line shows the regression predicted by the GLM and dashed lines indicate the 95% confidence interval.

To formally evaluate factors influencing PCR product polymorphism, we fitted two GLMs (see Materials and Methods for details). In the first of these, PCR product polymorphism was expressed as a binary factor, coded as 1 (polymorphic) or 0 (monomorphic). The minimum number of repeat units and *in silico* variability were both retained as highly significant predictor variables (*P*<0.0001) in the reduced model, which explained 60% of the total deviance (Table 3). This suggests that, even after controlling for a positive relationship between microsatellite length and polymorphism, loci that appear variable *in silico* were more likely to yield polymorphic PCR products. In the second GLM, we expressed PCR product polymorphism as the number of observed alleles and modeled this using a Poisson link function. The minimum number of repeat units and the number of alleles inferred from the 454 data were both retained as significant positive predictors (Table 4, Figure 2), whereas the number of reads differing from the consensus sequence was negatively correlated with the observed number of alleles. One potential explanation for the latter could be that loci showing many differences from the consensus sequence tend to possess fewer, but higher frequency alleles.

**Table 3:** Results of Generalized Linear Model (GLMs) of PCR product polymorphism (see Materials and Methods for details).

Term^a^	Estimate	χ^2^	df^b^	*P*
Minimum number of repeat units *in silico*	1.37	20.43	1	<0.0001
Variability *in silico*	8.23	17.32	1	<0.0001

a Only significant terms remaining in the reduced model are shown.

b Degrees of freedom.

Total deviance = 50.98; total explained deviance = 59.99%.

**Table 4:** Results of the Generalized Linear Model (GLM) of the number of alleles (see Materials and Methods for details).

Term^a^	Estimate	χ^2^	df^b^	*P*
Minimum number of repeat units *in silico*	0.06	3.87	1	0.049
Number of alleles *in silico*	0.34	6.88	1	0.009
Number of reads differing from consensus	−0.09	5.21	1	0.022

a Only significant terms remaining in the reduced model are shown.

b Degrees of freedom.

Total deviance = 24.37; total explained deviance = 57.07%.

## Discussion

Few if any studies have used cDNA to develop panels of candidate-gene targeted microsatellites in non-model organisms, while none to our knowledge have explored the possibility of screening microsatellites for variability *in silico*. Consequently, we used an Antarctic fur seal transcriptome assembly to develop PCR primers to amplify microsatellites residing within transcripts related to immunity and growth, while at the same time looking for a potential relationship between *in silico* variability and PCR product polymorphism. We were successful on both counts, identifying 13 polymorphic microsatellites associated with these two primary classes of candidate gene and also demonstrating a clear link between *in silico* variability and two different measures of PCR product polymorphism.

### Microsatellites within candidate transcripts

It has long been recognized that microsatellites derived from transcribed sequences, whether these be expressed sequence tags or transcriptome assemblies, can be powerful tools for a variety of applications including linkage and QTL mapping, comparative genomics and studies of genome evolution [39]. This is largely due to the fact that genes are often highly conserved, increasing the likelihood of primers cross-amplifying and serving as ‘anchor points’ for cross-species comparisons [40]. Being type I markers (i.e. associated with genes of known function), transcript-associated microsatellites also represent ideal markers for studying the genetic basis of phenotypic trait variation [13]. However, despite several studies having developed panels of candidate-gene targeted microsatellites from the complete genome sequences of humans and their companion species [41]–[43], cDNA has not yet been widely exploited for this purpose, particularly in non-model organisms. Our study highlights the eminent feasibility of such an approach, although the numbers of markers obtainable will clearly depend on the relative abundance of various classes of transcript. In this particular case, immune-related transcripts were relatively rare (total *n* = 316), perhaps unsurprisingly given that the transcriptome was developed from fur seal skin plugs [15]. Moreover, only 26 of these transcripts contained microsatellites, of which seven were discarded due to there not being enough flanking sequence to design primers and one because it did not map to the dog genome. Thus, the 13 primer pairs that we evaluated comprised the majority (72.2%) of available immune-related loci. In contrast, we only evaluated around a quarter of the 106 microsatellite-containing growth-related transcripts available to us. It should be possible to overcome the paucity of immune-related transcripts in this species through additional 454 sequencing directed towards other tissues such as the spleen. Alternatively, short read sequencing could be employed to increase the depth of coverage of singletons so far excluded from the assembly, a fraction of which would be expected to be related to immunity.

Notably, our rates of success in developing polymorphic loci were roughly equal for microsatellites located within immune and growth-related transcripts but higher for markers selected on the basis of *in silico* variability. This trend was also reflected in the allelic diversity of loci that successfully amplified. This is almost certainly due to our having been unbiased in our selection of immune and growth-related microsatellites, attempting to PCR amplify loci irrespective of the number of repeat units or apparent levels of variability. However, with a success rate of 80% for loci selected on the basis of high i*n silico* variability, our study suggests that *in silico* screening could significantly improve the efficiency of future attempts to develop candidate-gene targeted microsatellites (see below).

### Null alleles

An important drawback of microsatellites is the occurrence of non-amplifying alleles, which can arise when a mutation within one or both of the primer binding sites prevents primer annealing [36]. Null alleles are found in up to 30% of loci [36], [44] and can significantly impact paternity analyses, leading if unrecognized to the false exclusion of true parents and downstream to errors in pedigree reconstruction [38]. Consequently, we inspected the primer binding sites of both of the loci that significantly deviated from HWE in the direction of homozygosity excess. We found evidence of a SNP within the binding site of the reverse primer for Agt5, the locus carrying the highest null allele frequency. By implication, 454 sequence alignments might not only be useful for targeting polymorphic microsatellites, but also for designing primers that minimize the risk of null alleles being amplified. The latter could potentially be achieved by selecting only loci with a reasonably high depth of coverage at both of the primer annealing sites and which show no evidence of SNPs within these regions.

### Relationship between *in silico* variability and PCR product polymorphism

Although the advent of 454 sequencing has allowed much of the microsatellite isolation process to be streamlined, the primer evaluation step has seen few improvements other than the introduction of the M13 tailed system [45]. Unfortunately, very little can be done beyond careful PCR optimization to minimize the wastage of time and materials on loci that fail to amplify. However, we believe that pre-selecting markers for variability *in silico* may help to minimize the number of primer pairs that yield monomorphic PCR products, especially in species with low levels of genetic variability (e.g. [14], [46], [47]). Furthermore, even in ‘normal’ species, a small improvement in efficiency might bring about significant time and cost savings when attempting to develop large panels of markers for applications such as genetic mapping.

The concept of sourcing polymorphic genetic markers within sequence assemblies is by no means new, with several studies having previously mined 454 datasets for SNPs [e.g. 48], [49]. However, we are not aware of any studies that have extended the same approach to microsatellites. If anything, the opposite appears to be the case, with most studies having sequenced a single specimen, while others have deliberately filtered out identical reads in order to reduce the risk of developing the same locus more than once [10]–[12], [43]. Although these strategies make some sense when genetic variability is high, under normal circumstances including more than one individual in a 454 run is unlikely to be detrimental. On the contrary, the inclusion of multiple individuals should not only capture more of the standing genetic variation within a given population, but may also help to average out any effects arising from differences in template quality among individuals.

On a related point, some authors have speculated that it should be possible to enrich for polymorphic microsatellites by selectively testing only loci with large numbers of repeat units [8], [10]. This is because longer microsatellites tend to be more mutable due to an increased probability of slippage [50], [51]. However, current Sanger and 454 read length limitations mean that the longer the microsatellite, the less flanking sequence will be available for designing primers [8]. Moreover, microsatellites also appear to have an upper size limit, rarely attaining lengths in excess of a few tens of repeat units [52]. Consequently, longer microsatellites may be relatively hard to come by, depending on genome size and the scale of the sequencing effort [24], [53]. Our approach may help to mitigate both of these problems. First, assembling multiple 454 reads allows longer tracts of contiguous sequence to be obtained, thereby maximizing the amount of flanking sequence available for primer design. In the case of the fur seal 454 assembly [15], average isotig length was three times greater than average read length (854 bp versus 286 bp). Second, we were also able to demonstrate a highly significant positive relationship between *in silico* variability and PCR product polymorphism, even after controlling for the number of repeat units. This suggest that pre-screening for microsatellites appearing variable *in silico* could help to increase the yield of informative markers regardless of whether or not these are additionally selected on the basis of length.

An important caveat to the above is that our approach was only around 75% accurate at predicting whether or not PCR products were polymorphic. However, most of the observed discrepancies appear to be explicable. For example, the locus that did not appear to be variable *in silico* but which generated polymorphic PCR products had a depth of coverage of only 4 reads. With so few reads in total, it is possible that stochastic variation during clonal amplification and sequencing could have resulted in a single allele being preferentially sequenced. Similarly, almost all of the eight loci that were monomorphic despite appearing variable *in silico* were characterized by the ‘minor allele’ being of very low frequency (typically only 1–3 copies). These minority reads could potentially have resulted from sequencing error, consistent with the fact that 454 error rates are somewhat higher than those typically experienced with Sanger sequencing [54]. Alternatively, they could represent genuine low-frequency alleles that were by chance present among those individuals comprising the discovery panel but absent from those comprising the genotyping panel. This possibility cannot be dismissed, although all of the individuals originated from the same population and the latter comprised twice as many individuals as the former. Regardless of the exact mechanism, it would seem prudent to focus future efforts primarily upon microsatellites with high coverage and for which several reads differ from the consensus genotype.

We also extended our approach beyond simply testing whether or not a locus was polymorphic by correlating the number of alleles observed *in silico* with those obtained through PCR. A significant positive relationship was obtained, which was again robust to controlling statistically for microsatellite length. This could be partly due to ascertainment bias, the use of fewer individuals for microsatellite discovery than genotyping potentially having favoured the preferential discovery of common alleles. Nevertheless, selecting for microsatellite loci carrying multiple alleles *in silico* would appear to provide a means of maximizing the average variability of a panel of markers for a given development effort.

One potential issue relating to the selection of maximally polymorphic markers is that these may derive preferentially from regions of the genome experiencing balancing selection [55], [56] and could therefore generate misleading results in population genetic analyses. However, the ten markers we selected on the basis of high *in silico* variability showed no obvious pattern either in terms of genomic distribution or functional annotation (Table S1). For example, these loci map to 8 different chromosomes in the dog, and none locate to chromosome 12 which carries an obvious candidate for balancing selection, the MHC [57]. Clearly, in order to better understand the distribution of microsatellite variability across the genome, it would be desirable to evaluate many more markers. One option in the fur seal would be to screen these across multiple populations to identify *F*_st_ outliers as potential candidates for loci behaving non-neutrally [58].

### Wider applicability

To take full advantage of the high-throughput nature of next-generation sequencing requires fully automated data processing. Automating the *in silico* screening of isotigs for variable microsatellites would be a logical extension to the proof of principle that we present here, and might potentially be achieved by modifying a pre-existing microsatellite search tool, of which there are many [59], [60]. Several of these programs are also capable of designing primers or can link up with external primer selection tools such as Primer 3 [25] to further streamline marker development. Given a large enough pool of candidate microsatellites, automation might also help to minimize the amount of time spent on manual PCR optimization. For example, loci that fail to amplify under standard conditions could be discarded and other markers drawn from the pool to replace them. This could greatly facilitate the rapid development of polymorphic microsatellites for candidate gene studies or the construction of high-density genetic maps [59].

Although we have developed *in silico* screening using a transcriptome assembly, it might also be possible to extend the same approach to other situations in which homologous sequence reads are available from more than one individual. This may not apply to whole-genome 454 shotgun sequencing-based approaches due to the probability of sequencing 100 bp of the same genomic sequence almost certainly being too low [10]. However, it may prove possible to align multiple homologous sequences from reduced representation libraries. Moreover, 454 technology will continue to improve, with a single GS FLX+ run already being capable of generating 700 Mb of sequence data with an average read length of 700 bp. The potential also exists in the future to exploit increasing numbers of large-scale genomic databases [59], some of which (e.g. the 1000 Genomes project; http://www.1000genomes.org) already incorporate sequence data from multiple individuals.

Finally, financial and time factors also need to be taken into account. A clear consensus is already emerging that 454 sequencing is more cost-effective, requires less time to be spent in the wet lab and generates many more markers than traditional approaches based on the Sanger sequencing of enriched genomic libraries [8], [10], [12], [61]. Our study employed a full 454 run costing around £8000, which lies squarely within the range of commercial fees reported for developing around ten polymorphic loci [8]. However, using *in silico* mining, we were able to identify over 2000 microsatellites, many of which were associated with annotated transcripts, at no additional cost and with a negligible time investment [15]. This compares favourably with two previous development efforts in this species, which together generated a total of 53 clone sequences from which primers could be designed [62], [63] at the cost of several months of laboratory work. Finally, the rate of conversion into polymorphic microsatellites averaged over the two previous studies was 35.8%, significantly lower than the 80% rate obtained here for loci selected on the basis of high *in silico* variability (Fisher's exact test, *P* = 0.014). Consequently, targeting loci that are more likely to successfully convert should bring significant time savings as well as reducing expenditure on oligonucleotides.

### Conclusion

In this manuscript, we build upon previous studies that have used 454 sequencing to identify microsatellites both by developing markers within specific functional classes of transcript and by demonstrating a positive correlation between *in silico* and observed measures of microsatellite variability. We hope that the latter finding will stimulate further interest in the development and application of *in silico* screening approaches.

## Supporting Information

Table S1 Details of 50 *Arctocephalus gazella* isotigs containing microsatellite motifs.
(DOCX)

Table S2 Details of 50 putative fur seal microsatellite loci tested for PCR amplification in 24 unrelated *Arctocephalus gazella* individuals.
(DOCX)
