# Supplementary material for: A Novel Approach for Mining Polymorphic Microsatellite Markers In Silico
Source: PLoS One. 2011 Aug 10;6(8):e23283. doi: 10.1371/journal.pone.0023283 (PMC3154332; doi:10.1371/journal.pone.0023283)
Supplement: Table S1 — Details of 50 Arctocephalus gazella isotigs containing microsatellite motifs. (DOCX) [file pone.0023283.s001.docx]

| Locus | Isotig | | | | | Top BLAST hit (nr database) | | | Top BLAST hit (dog genome) | | |
| --- | --- | --- | --- | --- | --- | --- | --- | --- | --- | --- | --- |
|  | ID^a^ | No. of reads | Length (bp) | Chromosome in dog | GO annotation terms linked to immunity or growth^b^ | E-value | Bit score | Subject description | E-value | Bit score | Subject description |
| Agt1 | isotig02618 | 122 | 2184 | 10 | innate immune response, macrophage activation during immune response | 0 | 927 | hypothetical protein PANDA_010473 [Ailuropoda melanoleuca] | 0 | 925 | PREDICTED: similar to bactericidal/permeability-increasing protein-like 2 [Canis familiaris] |
| Agt2 | isotig02706 | 151 | 3134 | 10 | somatic hypermutation of immunoglobulin genes, somatic recombination of immunoglobulin gene segments | 5E-177 | 626 | hypothetical protein PANDA_000220 [Ailuropoda melanoleuca] | 8E-178 | 622 | PREDICTED: similar to mutS homolog 6 [Canis familiaris] |
| Agt3 | isotig05544 | 37 | 1008 | 2 | positive regulation of adaptive immune response | 6E-11 | 72.8 | interleukin 6 signal transducer (gp130, oncostatin M receptor) [Sus scrofa] | 5E-13 | 72.8 | PREDICTED: similar to Interleukin-6 receptor beta chain precursor (IL-6R-beta) (Interleukin 6 signal transducer) (Membrane glycoprotein 130) (gp130) (Oncostatin M receptor) (CDw130) (CD130 antigen) [Canis familiaris] |
| Agt4 | isotig05556 | 373 | 5144 | 4 | immune response | 0 | 895 | hypothetical protein PANDA_003412 [Ailuropoda melanoleuca] | 0 | 895 | PREDICTED: similar to bone morphogenetic protein receptor, type IA precursor isoform 7 [Canis familiaris] |
| Agt5 | isotig08633 | 46 | 826 | 25 | immune response | 2E-34 | 150 | chemokine (C-C motif) ligand 20 [Sus scrofa] | 1E-33 | 140 | chemokine (C-C motif) ligand 20 [Canis lupus familiaris] |
| Agt6 | isotig09106 | 10 | 708 | 14 | immune response | 5E-23 | 112 | PREDICTED: neural Wiskott-Aldrich syndrome protein-like [Callithrix jacchus] | 4E-25 | 112 | PREDICTED: similar to Wiskott-Aldrich syndrome gene-like protein isoform 1 [Canis familiaris] |
| Agt7 | isotig12147 | 40 | 681 | 2 | immune response | 4E-66 | 254 | PREDICTED: protein Red [Callithrix jacchus] | 4E-68 | 254 | PREDICTED: similar to RED protein [Canis familiaris] |
| Agt8 | isotig14698 | 33 | 811 | 17 | positive regulation of innate immune response | 8E-47 | 191 | PREDICTED: similar to polymerase (RNA) III (DNA directed) polypeptide G (32kD) like [Canis familiaris] | 7E-49 | 191 | PREDICTED: similar to polymerase (RNA) III (DNA directed) polypeptide G (32kD) like [Canis familiaris] |
| Agt9 | isotig16351 | 44 | 1752 | 7 | immunoglobulin biosynthetic process, regulation of humoral immune response mediated by circulating immunoglobulin, positive regulation of T cell mediated immunity, positive regulation of humoral immune response mediated by circulating immunoglobulin | 1E-157 | 561 | hypothetical protein PANDA_002581 [Ailuropoda melanoleuca] | 2E-152 | 536 | PREDICTED: similar to protein tyrosine phosphatase, receptor type, C isoform 3 precursor [Canis familiaris] |
| Agt10 | isotig16712 | 45 | 1128 | 32 | immune response | 5E-44 | 182 | chemokine C-X-C motif ligand 10 [Mustela putorius furo] | 9E-40 | 161 | C-X-C motif chemokine 10 precursor [Canis lupus familiaris] |
| Agt11 | isotig17268 | 35 | 998 | 7 | immune system development, regulation of immune response, immune response | 9E-41 | 171 | PREDICTED: mothers against decapentaplegic homolog 3 [Oryctolagus cuniculus] | 8E-43 | 171 | PREDICTED: similar to MAD homolog 2 isoform 5 [Canis familiaris] |
| Agt12 | isotig19423 | 71 | 991 | 34 | humoral immune response | 6E-134 | 481 | PREDICTED: similar to CMP-N-acetylneuraminate-beta-galactosamide-alpha-2,6-sialyltransferase (Beta-galactoside alpha-2,6-sialyltransferase) (Alpha 2,6-ST) (Sialyltransferase 1) (ST6Gal I) (B-cell antigen CD75) isoform 1 [Canis familiaris] | 4E-136 | 481 | PREDICTED: similar to CMP-N-acetylneuraminate-beta-galactosamide-alpha-2,6-sialyltransferase (Beta-galactoside alpha-2,6-sialyltransferase) (Alpha 2,6-ST) (Sialyltransferase 1) (ST6Gal I) (B-cell antigen CD75) isoform 1 [Canis familiaris] |
| Agt13 | isotig22789 | 180 | 1780 | 38 | immune response | 8E-125 | 452 | PREDICTED: similar to interferon-inducible protein 203 [Equus caballus] | 2E-79 | 294 | PREDICTED: similar to myeloid cell nuclear differentiation antigen [Canis familiaris] |
| Agt14 | isotig01178 | 141 | 740 | 25 | insulin-like growth factor receptor signaling pathway | 1E-74 | 283 | PERQ amino acid-rich with GYF domain-containing protein 2 isoform b [Mus musculus] | 2E-76 | 282 | PREDICTED: similar to trinucleotide repeat containing 15 isoform 1 [Canis familiaris] |
| Agt15 | isotig01475 | 1196 | 3729 | 10 | positive regulation of growth rate, growth | 1E-29 | 99.4 | hypothetical protein PANDA_002887 [Ailuropoda melanoleuca] | 2E-31 | 98.2 | PREDICTED: similar to prenylcysteine oxidase 1 [Canis familiaris] |
| Agt16 | isotig01600 | 1349 | 1258 | 15 | epidermal growth factor binding | 1E-45 | 188 | PREDICTED: nardilysin isoform 2 [Callithrix jacchus] | 1E-47 | 188 | PREDICTED: similar to Nardilysin precursor (N-arginine dibasic convertase) (NRD convertase) (NRD-C) isoform 2 [Canis familiaris] |
| Agt17 | isotig01608 | 610 | 2106 | 4 | growth | 9E-145 | 518 | selenoprotein P precursor [Canis lupus familiaris] | 6E-147 | 518 | selenoprotein P precursor [Canis lupus familiaris] |
| Agt18 | isotig02159 | 313 | 1021 | 30 | transforming growth factor beta receptor signaling pathway | 0 | 763 | hypothetical protein PANDA_005925 [Ailuropoda melanoleuca] | 0 | 759 | PREDICTED: similar to Fibrillin-1 precursor isoform 3 [Canis familiaris] |
| Agt19 | isotig02957 | 110 | 1855 | 28 | growth | 0 | 916 | hypothetical protein PANDA_003432 [Ailuropoda melanoleuca] | 0 | 916 | PREDICTED: similar to eukaryotic translation initiation factor 3, subunit 10 (theta) isoform 2 [Canis familiaris] |
| Agt20 | isotig03111 | 533 | 1325 | 17 | cell growth | 3E-125 | 452 | PREDICTED: similar to nucleophosmin 1 isoform 1 [Canis familiaris] | 2E-127 | 452 | PREDICTED: similar to nucleophosmin 1 isoform 1 [Canis familiaris] |
| Agt21 | isotig03503 | 68 | 1984 | 26 | growth | 1E-69 | 245 | PREDICTED: similar to ATPase family, AAA domain containing 1 [Canis familiaris] | 7E-72 | 245 | PREDICTED: similar to ATPase family, AAA domain containing 1 [Canis familiaris] |
| Agt22 | isotig03536 | 31 | 532 | 29 | regulation of growth hormone secretion, positive regulation of multicellular organism growth | 4E-33 | 144 | PREDICTED: similar to chromodomain helicase DNA binding protein 7 isoform 1 [Canis familiaris] | 5E-35 | 144 | PREDICTED: similar to chromodomain helicase DNA binding protein 7 isoform 1 [Canis familiaris] |
| Agt23 | isotig04211 | 181 | 3175 | 34 | positive regulation of growth rate, growth | 8E-150 | 536 | unnamed protein product [Mus musculus] | 4E-142 | 503 | PREDICTED: similar to RP42 homolog isoform 1 [Canis familiaris] |
| Agt24 | isotig04949 | 191 | 2239 | 9 | regulation of cell growth | 5E-55 | 220 | intraflagellar transport 20 homolog [Rattus norvegicus] | 4E-56 | 217 | PREDICTED: similar to intraflagellar transport protein IFT20 [Canis familiaris] |
| Agt25 | isotig04990 | 205 | 2760 | 3 | prostate gland growth | 0.00004 | 55.5 | PREDICTED: ubiquitin-protein ligase E3A isoform 3 [Callithrix jacchus] | 0.0000003 | 55.5 | PREDICTED: similar to ubiquitin protein ligase E3A isoform 1 isoform 1 [Canis familiaris] |
| Agt26 | isotig05465 | 125 | 2421 | 1 | positive regulation of cell growth | 0 | 799 | PREDICTED: similar to serum/glucocorticoid regulated kinase isoform 2 [Canis familiaris] | 0 | 799 | PREDICTED: similar to serum/glucocorticoid regulated kinase isoform 2 [Canis familiaris] |
| Agt27 | isotig05808 | 118 | 1748 | 37 | cell growth | 0 | 761 | nucleolar protein 58 [Homo sapiens] | 0 | 759 | PREDICTED: similar to Nucleolar protein NOP5 (Nucleolar protein 5) (NOP58) isoform 1 [Canis familiaris] |
| Agt28 | isotig06106 | 98 | 1328 | 6 | positive regulation of platelet-derived growth factor receptor signaling pathway | 1E-46 | 192 | coagulation factor III [Canis lupus familiaris] | 8E-49 | 192 | coagulation factor III [Canis lupus familiaris] |
| Agt29 | isotig06811 | 201 | 2353 | 32 | positive regulation of growth rate | 5E-58 | 221 | PREDICTED: similar to ELOVL family member 6, elongation of long chain fatty acids (FEN1/Elo2, SUR4/Elo3-like, yeast) isoform 3 [Macaca mulatta] | 5E-59 | 217 | PREDICTED: similar to ELOVL family member 6, elongation of long chain fatty acids (FEN1/Elo2, SUR4/Elo3-like, yeast) [Canis familiaris] |
| Agt30 | isotig10999 | 80 | 649 | 1 | positive regulation of growth rate, growth | 6E-53 | 211 | PREDICTED: similar to mitochondrial ribosomal protein L18 [Canis familiaris] | 6E-55 | 211 | PREDICTED: similar to mitochondrial ribosomal protein L18 [Canis familiaris] |
| Agt31 | isotig11118 | 9 | 583 | 2 | regulation of multicellular organism growth | 1E-30 | 136 | seven in absentia 1A-like [Bos taurus] | 1E-32 | 136 | PREDICTED: similar to Ubiquitin ligase SIAH1 (Seven in absentia homolog 1) (Siah-1) (Siah-1a) isoform 2 [Canis familiaris] |
| Agt32 | isotig11764 | 20 | 488 | 1 | nerve growth factor binding, nerve growth factor production | 1E-48 | 196 | PREDICTED: proprotein convertase subtilisin/kexin type 5 [Callithrix jacchus] | 3E-28 | 121 | PREDICTED: similar to Proprotein convertase subtilisin/kexin type 5 precursor (Proprotein convertase PC5) (Subtilisin/kexin-like protease PC5) (PC6) (Subtilisin-like proprotein convertase 6) (SPC6) [Canis familiaris] |
| Agt33 | isotig12362 | 180 | 1484 | 22 | platelet-derived growth factor binding | 1E-16 | 65.1 | procollagen, type IV, alpha 1, isoform CRA_b [Rattus norvegicus] | 2E-12 | 71.6 | PREDICTED: similar to Collagen alpha 1(IV) chain precursor [Canis familiaris] |
| Agt34 | isotig12464 | 69 | 1915 | 29 | positive regulation of cell growth | 6E-36 | 154 | hypothetical protein PANDA_004493 [Ailuropoda melanoleuca] | 5E-38 | 154 | PREDICTED: similar to Serine/threonine-protein kinase Sgk3 (Serum/glucocorticoid regulated kinase 3) (Serum/glucocorticoid regulated kinase-like) isoform 1 [Canis familiaris] |
| Agt35 | isotig14288 | 23 | 492 | 38 | regulation of cell growth | 3E-50 | 201 | PREDICTED: discoidin domain-containing receptor 2 isoform 1 [Callithrix jacchus] | 5E-51 | 197 | PREDICTED: similar to discoidin domain receptor family, member 2 precursor isoform 1 [Canis familiaris] |
| Agt36 | isotig15829 | 48 | 1190 | 18 | positive regulation of growth rate, growth | 9E-50 | 202 | PREDICTED: FACT complex subunit SSRP1 [Callithrix jacchus] | 3E-45 | 180 | PREDICTED: similar to structure specific recognition protein 1 isoform 1 [Canis familiaris] |
| Agt37 | isotig16846 | 136 | 3375 | 3 | platelet-derived growth factor receptor signaling pathway | 2E-128 | 437 | PREDICTED: similar to arrestin domain containing 3 isoform 1 [Canis familiaris] | 1E-130 | 437 | PREDICTED: similar to arrestin domain containing 3 isoform 1 [Canis familiaris] |
| Agt38 | isotig18325 | 95 | 1329 | 1 | growth factor activity, fibroblast growth factor receptor signaling pathway, insulin-like growth factor binding | 5E-49 | 127 | connective tissue growth factor (predicted) [Otolemur garnettii] | 1E-50 | 127 | PREDICTED: similar to connective tissue growth factor [Canis familiaris] |
| Agt39 | isotig19194 | 25 | 379 | 14 | growth cone | 5E-13 | 77.4 | unnamed protein product [Homo sapiens] | 5E-15 | 76.6 | PREDICTED: similar to Exocyst complex component Sec8 [Canis familiaris] |
| Agt40 | isotig22410 | 12 | 855 | 3 | prostate gland growth | 1E-122 | 322 | PREDICTED: similar to ubiquitin protein ligase E3A isoform 3 isoform 2 [Canis familiaris] | 9E-125 | 322 | PREDICTED: similar to ubiquitin protein ligase E3A isoform 3 isoform 2 [Canis familiaris] |
| Agt41 | isotig00949 | 99 | 740 | 17 | – | 7E-151 | 538 | PREDICTED: similar to Retinol dehydrogenase 12 [Canis familiaris] | 5E-153 | 538 | PREDICTED: similar to Retinol dehydrogenase 12 [Canis familiaris] |
| Agt42 | isotig01816 | 138 | 1596 | 15 | – | 2E-135 | 487 | PREDICTED: similar to oxysterol binding protein-like 9 isoform b isoform 6 [Canis familiaris] | 1E-137 | 487 | PREDICTED: similar to oxysterol binding protein-like 9 isoform b isoform 6 [Canis familiaris] |
| Agt43 | isotig06868 | 403 | 1152 | 17 | – | 6E-29 | 132 | hypothetical protein PANDA_021513 [Ailuropoda melanoleuca] |  |  |  |
| Agt44 | isotig16939 | 46 | 1133 | 15 | – | 4E-18 | 97.1 | LOC552889 protein [Homo sapiens] |  |  |  |
| Agt45 | isotig17192 | 138 | 3206 | 29 | – | 6E-61 | 241 | hypothetical protein PANDA_005084 [Ailuropoda melanoleuca] | 6E-71 | 222 | PREDICTED: similar to Carbonic anhydrase II (Carbonate dehydratase II) (CA-II) (Carbonic anhydrase C) isoform 2 [Canis familiaris] |
| Agt46 | isotig17879 | 130 | 2407 | 18 | – | 0 | 867 | hypothetical protein PANDA_001500 [Ailuropoda melanoleuca] | 0 | 865 | PREDICTED: similar to Nicotinamide phosphoribosyltransferase (NAmPRTase) (Nampt) (Pre-B-cell colony-enhancing factor 1 homolog) (PBEF) [Canis familiaris] |
| Agt47 | isotig18165 | 125 | 1223 | 16 | – | 1E-50 | 204 | PREDICTED: hypothetical protein XP_843762 [Canis familiaris] | 1E-52 | 204 | PREDICTED: hypothetical protein XP_843762 [Canis familiaris] |
| Agt48 | isotig20149 | 65 | 1865 | 31 | – | 8E-113 | 412 | hypothetical protein PANDA_002384 [Ailuropoda melanoleuca] | 1E-114 | 411 | PREDICTED: similar to Ubiquitin carboxyl-terminal hydrolase 25 (Ubiquitin thiolesterase 25) (Ubiquitin-specific processing protease 25) (Deubiquitinating enzyme 25) (USP on chromosome 21) [Canis familiaris] |
| Agt49 | isotig20932 | 41 | 1322 | 30 | – | 1E-24 | 119 | PREDICTED: similar to small nuclear RNA activating complex, polypeptide 5, 19kDa [Canis familiaris] | 8E-27 | 119 | PREDICTED: similar to small nuclear RNA activating complex, polypeptide 5, 19kDa [Canis familiaris] |
| Agt50 | isotig21434 | 79 | 1284 | 2 | – | 1E-143 | 514 | PREDICTED: similar to plexin domain containing 2 precursor [Canis familiaris] | 9E-146 | 514 | PREDICTED: similar to plexin domain containing 2 precursor [Canis familiaris] |

a Short read sequences are available via Genbank (accession number ERP000497) and assembled isotig sequences via Dryad (doi 10.5061/dryad.8268).

b Only GO annotations relating to immunity or growth are listed.
