# Supplementary material for: A Novel Approach for Mining Polymorphic Microsatellite Markers In Silico
Source: PLoS One. 2011 Aug 10;6(8):e23283. doi: 10.1371/journal.pone.0023283 (PMC3154332; doi:10.1371/journal.pone.0023283)
Supplement: Table S2 — Details of 50 putative fur seal microsatellite loci tested for PCR amplification in 24 unrelated Arctocephalus gazella individuals. (DOCX) [file pone.0023283.s002.docx]

| Locus^a^ | Sequence ID^b^ | Repeat motif | Depth of coverage^c^ | No. of reads differing from consensus | No. of alleles^d^ | Forward primer sequence (5'–3') | Reverse primer sequence (5'–3') | Expected product size (bp) | PCR products^e,f^ |
| --- | --- | --- | --- | --- | --- | --- | --- | --- | --- |
| Agt1 | isotig02618 | AG_5_ | 12 | 0 | 1 | TGAACCAAATGGCTGGAAAT | GGCGTATTCTACCCACTGGA | 197 | – |
| Agt2 | isotig02706 | TCC_5_ | 5 | 0 | 1 | CTAACTGGCCAGCCTGTCTC | GTTGCCAGAAGAAGGCACTC | 221 | – |
| Agt3 | isotig05544 | AT_5_ | 15 | 0 | 1 | ATCAACCAAATTCTCCAGCA | CAAAATTTGCTTTCGGTCGT | 186 | Monomorphic |
| Agt4 | isotig05556 | GT_3_AC_5_ | 13 | 0 | 1 | TGAATTCAGCAGAGATGGAGAA | AATTCAAATGGCAGCAGAGG | 201 | Monomorphic |
| Agt5 | isotig08633 | AC_4_(AAA)AC_3-4_AT_5-8_ | 16 | 8 | 3 | TTGCTGCTTACAAGAAAGTCCA | CGATTTGTGCTTCGTTGGAT | 246 | Polymorphic |
| Agt6 | isotig09106 | AT_6_(A)AT_3_ | 4 | 0 | 1 | CTTGCACACAATAACAGGGAGT | GGATGATGATGAATGGGAAGA | 242 | Monomorphic |
| Agt7 | isotig12147 | CT_7-8_ | 5 | 1 | 2 | GCCCACAGCCCTGTAGTTAG | GTCACGTCACCATGAGATGC | 229 | – |
| Agt8 | isotig14698 | CTT_4-5_ | 9 | 3 | 2 | CCAAAGTCCTCTCCGTTGTC | GCTACAAAAGGAACGGACCA | 249 | – |
| Agt9 | isotig16351 | CT_6-7_ | 9 | 3 | 2 | AAAGGGTAAAGTTTCTCAAAAGCA | TTGGACCAATTTTTGAGCAG | 240 | Polymorphic |
| Agt10 | isotig16712 | AT_9-11_ | 10 | 4 | 3 | AAGGGGCCCATATTCTTCC | CATTGCACAGTCATGTGTGG | 213 | Polymorphic |
| Agt11 | isotig17268 | AC_5_ | 14 | 0 | 1 | GACGCACAAAAATAACGGAGA | TGGGCATAAAACTGAGTGTCC | 219 | Monomorphic |
| Agt12 | isotig19423 | GT_4_(AT)GT_3-5_ | 28 | 1 | 2 | GGTGGGAAAGGTGATGTGAA | AGCCCTTCTCCACCAAGTCT | 226 | – |
| Agt13 | isotig22789 | AT_4-5_GA_3_ | 6 | 1 | 2 | AAACCTGGACGCCATTAACA | GCCATAGGATGGCACAGAAA | 175 | Polymorphic |
| Agt14 | isotig01178 | GCA_5-6_ | 34 | 5 | 2 | CTCTGAGGCCAAGGAGTTTG | TCGGACCATCTTCTGCTTTT | 214 | – |
| Agt15 | isotig01475 | AT_6-8_ | 20 | 10 | 3 | TGAAAAACAGAACACAATGGATG | TTATTCCCTTCTCCAGCAGC | 173 | – |
| Agt16 | isotig01600 | TCA_4-5_ | 6 | 2 | 2 | GCCCTCTCTTCTAATTCTTCCA | AAACAGGAGATGCAACAGATGA | 232 | Polymorphic |
| Agt17 | isotig01608 | CAC_6-7_ | 78 | 3 | 2 | ATGACCCTGGAAGCTGAAGA | TGTGTTTATGGTGGCAATGG | 232 | Monomorphic |
| Agt18 | isotig02159 | GT_5_(TG)AC_4_ | 34 | 0 | 1 | GAAACACCTGGGATCTGCAT | TGACACACTGCCCTCTCTTG | 163 | Monomorphic |
| Agt19 | isotig02957 | CT_5_ | 10 | 0 | 1 | TGACCCGCTCCTGATACTCT | GGCATAATCGCTTGGAAGAA | 186 | – |
| Agt20 | isotig03111 | ATC_5-6_(TCC)ATC_4-5_ | 114 | 8 | 2 | TTGGTGTTGACGGTTTTGAG | CTGCTCCTGGAAGTGGTAGC | 234 | Polymorphic |
| Agt21 | isotig03503 | AC_13_ | 4 | 0 | 1 | CACCGTACCTTAGACACAACCA | ACGTTGCAGCCTTAGAGCAT | 247 | Polymorphic |
| Agt22 | isotig03536 | AGA_5-7_ | 18 | 2 | 3 | TGGACTCCTCTTGGAACCAG | CTGGGATCCTCTGCAACAAT | 206 | – |
| Agt23 | isotig04211 | AT_5-6_ | 9 | 2 | 2 | CGAATTAATCAATCCGAAAACA | CCTGCTGCTTTCAGTATTTCC | 152 | Polymorphic |
| Agt24 | isotig04949 | GT_11-15_ | 28 | 15 | 5 | GTGGTCAGCAGGGACCTTTA | ATGCAGGGTCTCTTCACTGG | 230 | Polymorphic |
| Agt25 | isotig04990 | TTG_6-7_ | 13 | 2 | 2 | ACTGCAGCCCTCACAACTTT | CAAATGCACTTTTCCCCAGT | 202 | Polymorphic |
| Agt26 | isotig05465 | CA_13-15_ | 7 | 4 | 3 | AGCACCATGTTGGAAGGAAG | GGGGTGGTCTTGAAGGATTT | 245 | Polymorphic* |
| Agt27 | isotig05808 | CTT_7-9_ | 30 | 9 | 3 | TCAGAAAGCGGTTCTTCCTT | ATTCCACACTGCCAACCTGT | 250 | – |
| Agt28 | isotig06106 | AT_5-7_ | 21 | 2 | 2 | TAATGCTGTCGTGGAAACCA | GCCCACGACGGAGTATATGT | 171 | Monomorphic |
| Agt29 | isotig06811 | CT_4_(TT)CT_6-9_ | 6 | 2 | 3 | TGTATGTGCTCGCACAGTTTC | GAAACCCCATTCCCTCCTTA | 209 | Polymorphic* |
| Agt30 | isotig10999 | TG_6-7_ | 38 | 10 | 2 | TCTGGAAGAACAGCCAGCTT | TGTCCAAGAGAGGGGAAGAA | 153 | Monomorphic |
| Agt31 | isotig11118 | AT_5-6_ | 4 | 1 | 2 | TGGTAGGTGGAAGCTAGACACA | CGGCAAACACACAAAACAAA | 248 | Monomorphic |
| Agt32 | isotig11764 | CT_3_(TTTT)CT_5-6_ | 14 | 2 | 2 | TGAGCGGCCATCTTAGATTT | AGTTCACCATCTCCCTGCAC | 164 | Polymorphic |
| Agt33 | isotig12362 | AT_5-7_ | 25 | 3 | 3 | AAACAGCCATCACGTAACCA | TTGACCTGCCTAATTGCTGA | 236 | Monomorphic |
| Agt34 | isotig12464 | AC_5_ | 2 | 0 | 1 | ATGGTTTCTGGATGGCAAAC | TGTGGCAGGACCAGATGATA | 198 | Monomorphic |
| Agt35 | isotig14288 | CT_5_ | 7 | 0 | 1 | TCCAGGAAATCCACCTTCTG | TGGGTCGGGAATAGAGAGTG | 227 | Monomorphic |
| Agt36 | isotig15829 | GAA_5-6_ | 13 | 1 | 2 | GATGCCAGGAGGGAATATGA | TCCGCCTTCTCTTCTTTTTG | 250 | – |
| Agt37 | isotig16846 | AT_4-5_ | 6 | 2 | 2 | TCATTTTTGCTGGTGCAGAG | TCTCATTCCAGCACACGTTC | 183 | Monomorphic |
| Agt38 | isotig18325 | AT_6-8_AC_4_ | 23 | 11 | 3 | CAAACGAAAAGGCACAAACA | CTGAGGCTGGGTCAAGTTCT | 242 | Polymorphic |
| Agt39 | isotig19194 | ATTT_5-6_ | 22 | 2 | 2 | TGAAGCCCTAAAAACAGAACAA | TAATTGAAGGGGGAGGAACC | 239 | Polymorphic |
| Agt40 | isotig22410 | CT_6_ | 3 | 0 | 1 | TCTGTACCAATGCCTCAGCA | AGGTGCCCCTAACAACTCCT | 204 | – |
| Agt41 | isotig00949 | GT_15-18_ | 26 | 4 | 3 | CCTTGTTCAGGGTTAGGAAAA | CAAGCCAAGCAACAAGATCA | 220 | Polymorphic |
| Agt42 | isotig01816 | AC_7-10_AT_6-9_AC_6-9_AT_4-5_ | 27 | 17 | 5 | ATCCCGGTCTTAAACCTTGC | TGTCATAGGTGAGGGCATGA | 239 | Polymorphic |
| Agt43 | isotig06868 | ACC_4-5_ | 134 | 8 | 2 | CGAACCTCAAAACATGCTGA | CCCAGACATCCCATCTCATT | 250 | Monomorphic |
| Agt44 | isotig16939 | AC_8-10_ | 12 | 6 | 3 | GAAAAAGCCACAAACCACAAA | GGAACTCTCCCTTTCCCTACC | 178 | Polymorphic |
| Agt45 | isotig17192 | AC_7-9_ | 4 | 2 | 2 | TAGCGGTTCCATACCCAAAG | TGGTGTATTGGAGGTGGTCA | 151 | Polymorphic |
| Agt46 | isotig17879 | GT_5-7_ | 13 | 3 | 3 | TCAAGAATGGGAAGGTGACA | AAACACAAACCCCCACACAT | 216 | Monomorphic |
| Agt47 | isotig18165 | GTTT_7-10_ | 28 | 14 | 4 | CTGCTGTCGTCACTGCACTT | CAGCATGGTCCAAGGAAGAC | 235 | Polymorphic |
| Agt48 | isotig20149 | GT_10-14_ | 11 | 8 | 5 | TGCAAAACATTGTCCTCCAT | TGAAGTGAGCGGCTGATATTT | 237 | Polymorphic |
| Agt49 | isotig20932 | TCC_6-8_ | 10 | 2 | 3 | CTTGTAAAGCAGGCCAGAGG | CACTGGAGTTGAGCACAAGG | 183 | Polymorphic |
| Agt50 | isotig21434 | GT_10-15_(TT)GT_5-6_ | 18 | 11 | 6 | CAGGCTACAGCAGCTTAGGG | TTCGAAGAGGCTCTGGTCAT | 224 | Polymorphic |

a Loci Agt1 to Agt13 and Agt14 to Agt40 inclusive were selected on the basis of having immune- or growth-related GO annotation terms respectively. Agt41 to Agt50 were tested because they appeared highly variable *in silico*.

b Short read sequences are available via Genbank (accession number ERP000497) and assembled isotig sequences via Dryad (doi 10.5061/dryad.8268).

c Only reads spanning the full length of the repetitive region were counted.

d Refers to the number of sequence variants within the 454 assembly that differ in the total number of repeats.

e '–' indicates PCR reactions that failed to generate interpretable products.

f '*' refers to PCR products that were clearly polymorphic but which could not be reliably scored due to the apparent co-amplification of a second locus.
